# A large-scale screening identified in *USH2A* gene the P3272L founder pathogenic variant explaining familial Usher syndrome in Sardinia, Italy

**DOI:** 10.1186/s12886-024-03578-4

**Published:** 2024-07-23

**Authors:** Rita Serra, Vincenzo Rallo, Maristella Steri, Stefania Olla, Maria Grazia Piras, Michele Marongiu, Myriam Gorospe, David Schlessinger, Antonio Pinna, Edoardo Fiorillo, Francesco Cucca, Andrea Angius

**Affiliations:** 1 Institute of Genetic and Biomedical Research, National Research Council (CNR), Cittadella Universitaria Di Cagliari, C/O S.S 554 Bivio Per Sestu Km 4, 500, 09042 Monserrato, CA Italy; 2 Department of Biomedical Sciences, University of Sassari, Sassari, Italy; 3 Laboratory of Genetics and Genomics, National Institute On Aging, Baltimore, MD USA; 4 Department of Medicine, Surgery and Pharmacy Ophthalmology Unit, University of Sassari, Sassari, Italy

**Keywords:** Usher syndrome, USH2A gene, Pathogenic variant, Sardinia Founder effect, Molecular screening

## Abstract

**Background:**

Usher syndrome (USH) encompasses a group of disorders characterized by congenital sensorineural hearing loss (SNHL) and retinitis pigmentosa (RP). We described the clinical findings, natural history, and molecular analyses of USH patients identified during a large-scale screening to identify quantitative traits related to ocular disorders in the SardiNIA project cohort.

**Methods:**

We identified 3 USH-affected families out of a cohort of 6,148 healthy subjects. 9 subjects presented a pathological phenotype, with SNHL and RP. All patients and their family members underwent a complete ophthalmic examination including best-corrected visual acuity, slit-lamp biomicroscopy, fundoscopy, fundus autofluorescence, spectral-domain optical coherence tomography, and electrophysiological testing. Audiological evaluation was performed with a clinical audiometer. Genotyping was performed using several arrays integrated with whole genome sequence data providing approximately 22 million markers equally distributed for each subject analyzed. Molecular diagnostics focused on analysis of the following candidate genes: *MYO7A, USH1C, CDH23, PCDH15, USH1G, CIB2, USH2A, GPR98, DFNB31, CLRN1,* and *PDZD7*.

**Results:**

A single missense causal variant in *USH2A* gene was identified in homozygous status in all patients and in heterozygous status in unaffected parents. The presence of multiple homozygous patients with the same phenotypic severity of the syndromic form suggests that the Sardinian USH phenotype is the result of a founder effect on a specific pathogenic variant related haplotype. The frequency of heterozygotes in general Sardinian population is 1.89. Additionally, to provide new insights into the structure of usherin and the pathological mechanisms caused by small pathogenic in-frame variants, like p.Pro3272Leu, molecular dynamics simulations of native and mutant protein–protein and protein–ligand complexes were performed that predicted a destabilization of the protein with a decrease in the free energy change.

**Conclusions:**

Our results suggest that our approach is effective for the genetic diagnosis of USH. Based on the heterozygous frequency, targeted screening of this variant in the general population and in families at risk or with familial USH can be suggested. This can lead to more accurate molecular diagnosis, better genetic counseling, and improved molecular epidemiology data that are critical for future intervention plans.

**Trial registration:**

We did not perform any health-related interventions for the participants.

**Supplementary Information:**

The online version contains supplementary material available at 10.1186/s12886-024-03578-4.

## Introduction

With a worldwide prevalence of 4 to 17 per 100,000 individuals, Usher syndrome (USH) accounts for more than 50% of all cases of combined vision and hearing loss [1].

USH encompasses a group of disorders characterized by congenital sensorineural hearing loss (SNHL) and retinitis pigmentosa (RP). Clinically, RP presents with progressive pigmentary degeneration of rod and cone photoreceptors and the ophthalmoscopic triad of pigmentary changes (bony spicules) at the mid-periphery, pale waxy optic disc, and generalized attenuation of arterioles, which result, firstly, in visual field constriction followed by severe vision loss [2].

Clinically, patients were initially divided into two different groups: one characterized by profound deafness, the other by moderate/severe hearing loss [3]. In 1994, Smith et al. [4] classified USH into three different types (1, 2, and 3), based on age of onset, severity, and progression of auditory, visual, and vestibular symptoms [4].

USH has been considered a monogenic, but genetically heterogeneous disease. Type 1 (USH1), approximately 30% of all USH patients, is the most severe form, with profound SNHL and vestibular dysfunction from birth, as well as progressive RP [5, 6]. The more common type 2 (USH2), approximately 50% of cases [7], show moderate/severe SNHL, normal vestibular function, and RP with onset at puberty. Type 3 (USH3) is the rarest form [8]. Patients with USH3 develop variable levels of hearing, vision, and vestibular impairment over time [8]. Hearing loss occurs before visual symptoms in all three subtypes. Fourteen USH loci have been mapped to date: nine for USH1, three for USH2, two for USH3, one USH modifier and one atypical USH gene. However, some cases cannot be attributed to these genes and are classified as atypical USH. Several studies in mice and humans have shown a digenic inheritance of deafness caused by pathogenic variant in USH genes and USH modifier *PDZD7* gene [9, 10].

The purpose of our study was to describe the clinical findings, natural history, and molecular analysis of the USH-affected families living in Sardinia, Italy.

## Methods

### Recruitment of USH patients

The study was conducted in accordance with the code of ethics of the World Medical Association (Declaration of Helsinki) for research involving human subjects and all participants gave informed consent to study protocols, which were approved by the Sardinian local research committee Ethical Committee of ASSLL of Sassari (2171/CE). USH patients were identified during a large-scale screening to identify quantitative traits related to eye disorders in the SardiNIA project cohort [11]. Here we used a large cohort of 6,148 healthy Sardinians from the SardiNIA project to study a spectrum of quantitative traits, some of which are related to ocular disorders. Based on this analysis, we showed that the prevalence of eye disorders like age-related macular degeneration (AMD) in SardiNIA cohort was significantly lower than data reported in major epidemiological studies on this topic in North America, Northern Europe, and Italy [12].

We identified 3 families for a total of 22 subjects, of whom 9 presented a diagnosis of Usher syndrome. However, only five of them were available to undergo further specific analysis. Therefore, USH patients and their available family members were extensively studied from an ophthalmological and a molecular point of view at the Institute of Genetic and Biomedical Research of the National Research Council (CNR). Our clinical hypotheses and previous phenotypic findings indicated a suspected USH2.

### Clinical examination

All individuals underwent a complete ophthalmic examination, including best-corrected visual acuity (BCVA) measured with Early Treatment for Diabetic Retinopathy Study (ETDRS) charts, slit lamp biomicroscopy, fundoscopy, fundus autofluorescence (FAF), spectral domain–optical coherence tomography (SD-OCT; Spectralis; Heidelberg Engineering), and electrophysiological testing.

Audiologic evaluation was performed using a clinical audiometer (Otometrics, Madsen, model Xeta 2, Denmark) according to the reference manual. Tonal audiometry in the frequency range 0.25–8 kHz and higher frequencies at 10 and 12.5 kHz were tested. The severity of hearing loss was classified as mild (20–40 dB), moderate (41–70 dB), severe (71–90 dB), or profound (> 91 dB).

### Molecular screening

According to the SardiNIA project, genotyping was performed using four different Illumina arrays (OmniExpress, ImmunoChip, Cardio-MetaboChip, and ExomeChip), providing a scaffold of 890,542 autosomal single nucleotide polymorphisms (SNPs) and 16,325 unique X-linked SNPs across the genome. In parallel, whole genome shotgun sequence data from other 3,514 Sardinian individuals were also available [11]. To increase genetic map resolution, genotypes were integrated with information provided by sequencing data using statistical inference methods. In greater detail, genotypes were firstly phased with MACH software, using 30 iterations of the Markov haplotyping chain and 400 states per iteration; secondly, all variants included in the sequencing panel were imputed with minimac software. This strategy allowed the availability of approximately 22 million markers for analysis in the SardiNIA dataset.

Molecular diagnostics focused on finding causative variants for USH were based on whole sequence analysis of the following selected candidate genes: *MYO7A, USH1C, CDH23, PCDH15, USH1G, CIB2, USH2A, GPR98, DFNB31, CLRN1,* and *PDZD7*.

Using the UCSC Table Browser web software, a BED (Browser Extensible Data) file containing the genomic location (chromosome, initial and final genomic coordinates) of the focused regions was produced. Bedtools intersect software was used to select variants located within the regions of interest.

Ensembl's VEP (Variant Effect Predictor) software was adopted to annotate the types of variants (SNPs, insertions, deletions, duplications, CNVs, or structural variants) by adding for each of them information based on reference databases (dbSNP, gnomAD,1000 GENOME) [13–15].

Selection of gene variants was based on minor allele frequency (MAF) < 1% and in silico prediction of strong potential impact on phenotype (stop gain, missense, frameshift, splice, stop loss, start loss, etc.) of coding or regulatory variants. Functional predictions for amino acid changes were conducted according to several models (SIFT, Polyphen).

USH shows an autosomal recessive transmission mode, therefore, variants were filtered for this pattern of inheritance. Sanger sequencing confirmed in all family members the results found by whole sequence analysis.

### Haplotype reconstruction

Linkage disequilibrium (LD) block construction and haplotype population frequency estimation was performed via Haploview 4.2 [16]. For accurate haplotype determination, from 3,514 samples in the initial cohort, a representative sample of 1,454 unrelated individuals were pooled using bcftools 1.7 [17]. We selected a genomic interval of approximately 30 kb, where the causal variant rs764182950 was located, through PLINK v1.90 [18]. Block size definition was carried out using the default algorithm “confidence intervals”. We set the standard parameters by including all markers independently of the MAF value and examined haplotypes exceeding frequencies above 0.9% to detect the pathogenic variant related to USH.

### Molecular modeling (Homology models and Free energy calculation)

The Iterative Threading ASSEmbly Refinement (I-TASSER) server (https://zhanglab.ccmb.med.umich.edu/) was utilized to build the models using default settings [19]. Pymol (https://pymol.org/) was used to display, analyze the built Usherin model, and built the mutate protein. To calculate the free energy, we used the following tools using the 3D structure of the usherin protein domain 3210–3402. DUET consolidates two complementary approaches mCSM and SDM in a consensus prediction, obtained by combining the results of the separate methods in an optimized predictor using Support Vector Machines (SVM) [20, 21]. Mupro developed two machine learning methods (Support Vector Machines and Neural Networks) and for the calculation we used the amino acid structure of the protein [22].

## Results

**Fig. 1:**
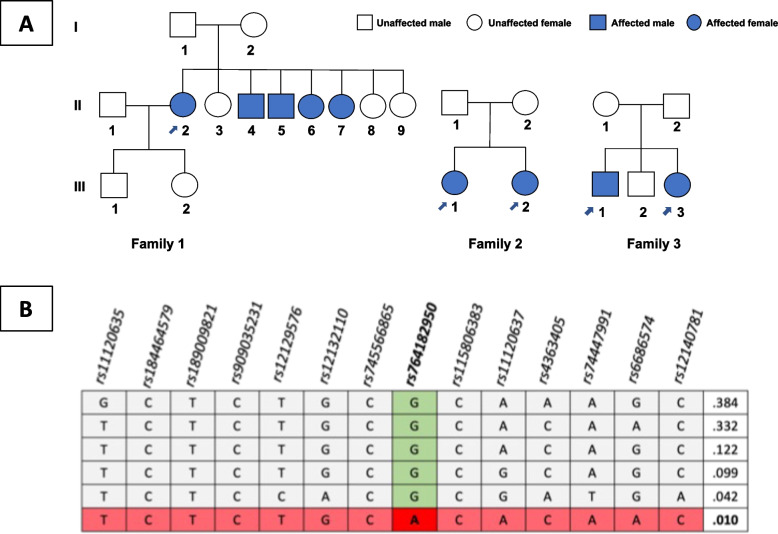
Clinical and genetics features of the three Sardinian pedigrees with members affected by Usher Syndrome. **A** In pedigree 1, we were able to obtain clinical/phenotypic information for three generations, but only for two generations in family 2 and 3. Patients indicated by the arrow were genetically and clinically re-evaluated. **B** Haplotype reconstruction using Haploview. Vertical green column shows the reference allele, horizontal red row indicates the haplotype nucleotide sequence. The alternative allele of the causal variant is shown in red and bold. Values on the right indicate the percentages of each haplotype

Five patients (4 women, 1 man; age range: 38–67 years) and 11 healthy relatives from three Sardinian families living in Lanusei valley were clinically and genetically examined (Fig. 1).

All patients complained of nyctalopia as initial symptom, occurred during adulthood. Night blindness was followed by gradual vision loss and constriction of peripheral vision in the late stages of the disease. Best-corrected visual acuity ranged from 40 to 100 ETDRS letters.

Fundus examination showed typical RP features including pigmentary changes in the peripheral and mid-peripheral retina, attenuated arteriolar vessels, and pallor of the optic disc.

Fundus autofluorescence revealed a macular hyper-autofluorescent ring around the fovea and hypo-autofluorescence within and extending outward of the vascular arcades.

**Fig. 2:**
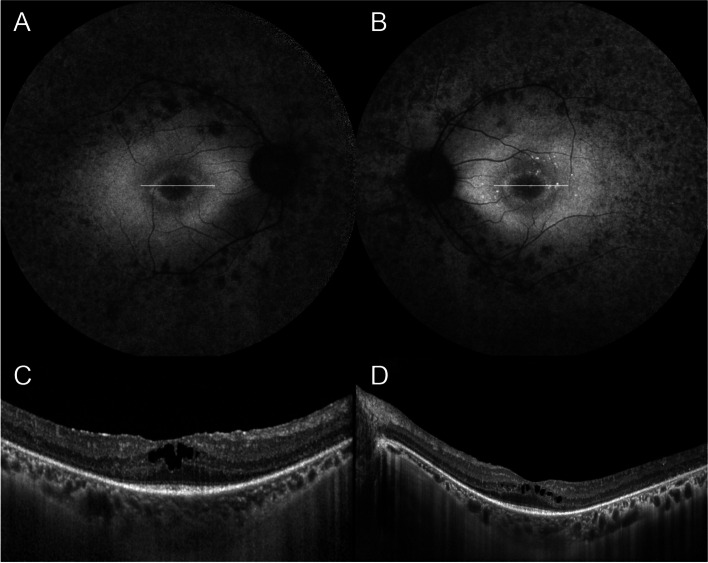
Multimodal imaging of a 38-year-old patient with Usher Syndrome. **A**, **B** Fundus autofluorescence revealed a macular hyperautofluorescent ring around the fovea and hypoautofluorescence within and extending outward of vascular arcades, bilaterally. **C**, **D** In both eyes, simultaneous infrared and SD-OCT scans demonstrated outer retinal atrophy with centrally preserved photoreceptor inner segment ellipsoid bands. Note the presence of bilateral intraretinal cystoid spaces in the foveal area (see white line in **A** and **B**)

SD-OCT scans demonstrated outer retinal atrophy with centrally preserved photoreceptor inner segment ellipsoid bands (Fig. 2).

Moreover, cystoid macular edema (CME) not responsive to topical and oral carbonic anhydrase inhibitors was detected in 3 eyes. None of the five individuals affected had vestibular abnormalities. Summary of demographics and clinical findings of all patients are shown in Table 1.

**Table 1:** Demographic and clinical findings of Usher patients

**ID**	**Gender**	**Age**	**Disease diagnosis**	**BCVA**^a^ **RE / LE**^b^	**CME**^c^ **RE / LE**
Family 1 II 2	Female	61	25	40 / 50	±
Family 2 III 1	Female	58	26	95 / 95	- / -
Family 2 III 2	Female	57	25	95 /95	- / -
Family 3 III 3	Female	38	19	95 / 90	+ / +
Family 3 III 1	Male	43	20	100 / 100	- / -

^a^*BCVA* Best Corrected Visual Acuity (ETDRS letters)

^b^*RE* Right Eye, *LE* Left Eye

^c^*CME* Cystoid Macular Edema

**Fig. 3:**
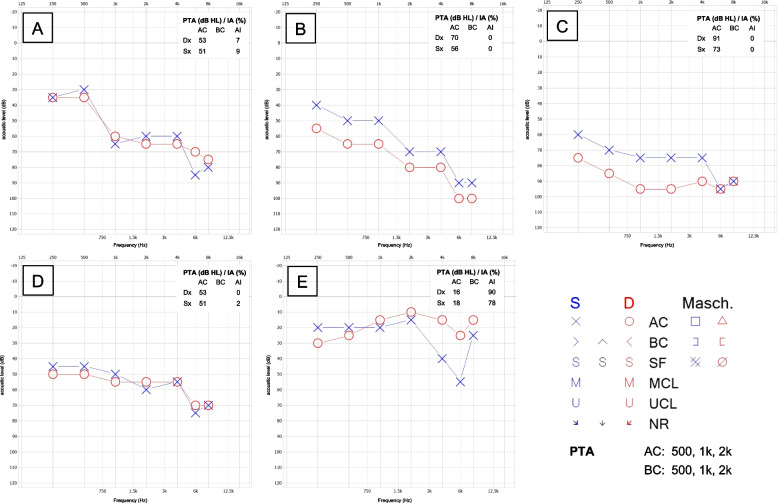
Audio profiles of Usher syndrome type 2A patients. The hearing loss of patient’s test (FAM-1 II.2; **A**, FAM-2 III.1; **B**, FAM-2 III.2, **C**, FAM-3 III.3, **D**) was performed using Otometrics, Madsen Xeta 2 clinical audiometer. We added a healthy control subject from family 3 (FAM-3 III.2, **E**)

Audiologic evaluation disclosed bilateral profound/severe hearing loss in patients FAM-2 II.1 (Dx 91 dB/ Sx 73 dB) and FAM-2 II.2 (Dx 70 dB/ Sx 56 dB). Moderate hearing loss was found in the remaining 3 patients (FAM-1 II.2 Dx 53 dB/ Sx 51 dB; FAM-3 III.3 Dx 53 dB/ Sx 51 dB). Healthy relatives showed normal values (for example FAM-3 III.2 Dx 16/ Sx 18 dB) (Fig. 3).

We selected homozygous variants for the alternative allele (both alleles mutated) and/or compound heterozygotes for two heterozygous alleles in patients versus reference homozygotes (i.e., both alleles wild type) and single heterozygotes (only one allele mutated) in healthy individuals in all coding sequences of the candidate genes.

Based on autosomal recessive genetic inheritance, we do not identify candidate pathogenic variant in the selected candidate genes, except for the *USH2A* gene. We found several variants in the *USH2A* gene (Supplementary Table 1), but only two of them were predicted to be pathogenic: c.9815C > T(p.Pro3272Leu) and c.1663C > G(p.Leu555Val). Based on the autosomal recessive model of inheritance, we identified a single potentially causative variant (p.Pro3272Leu) on the gene encoding for the usherin protein.

This pathogenic variant causes base substitution in an exonic region of the gene. The functional predictions for amino acid changes according to SIFT and Polyphen were *“probably damaging”* and *“deleterious”*, respectively, while the CADD score (CADD PHRED = 28,6) confirmed its pathogenicity. The American College of Medical Genetics and Genomics classification identifies this variant as probably pathogenic, but several studies prove in both homozygosity and heterozygosity status [23–25] the pathogenicity of this variant. Furthermore, the frequency of this variant in the gnomAD database, in non-Finnish European countries, is extremely rare (0.00004406%), in agreement with syndrome frequencies. All patients analyzed were found to be homozygous for the c.9815C > T variant. Since USH is a rare syndrome, several patients from the same geographical area affected by the same pathogenic variant imply a probable founder effect, proven by the identification of an extended haplotype using subjects from all areas of Sardinia. The frequency of c.9815C > T (p.Pro3272Leu) heterozygous subjects was estimated to be 1,89%. Pathogenic variant related haplotype reconstruction in unrelated Sardinian sequenced samples revealed a founder effect (Fig. 1). Haplotype analysis surrounding the causal SNP, rs764182950, indicated that the haplotype “TCTCTGCACACAAC” consisting of 14 variants (rs11120635, rs184464579, rs189009821, rs909035231, rs12129576, rs12132110, rs745566865, rs764182950, rs115806383, rs11120637, rs4363405, rs74447991, rs6686574, rs12140781) represents a risk factor for USH in Sardinia.

**Fig. 4:**
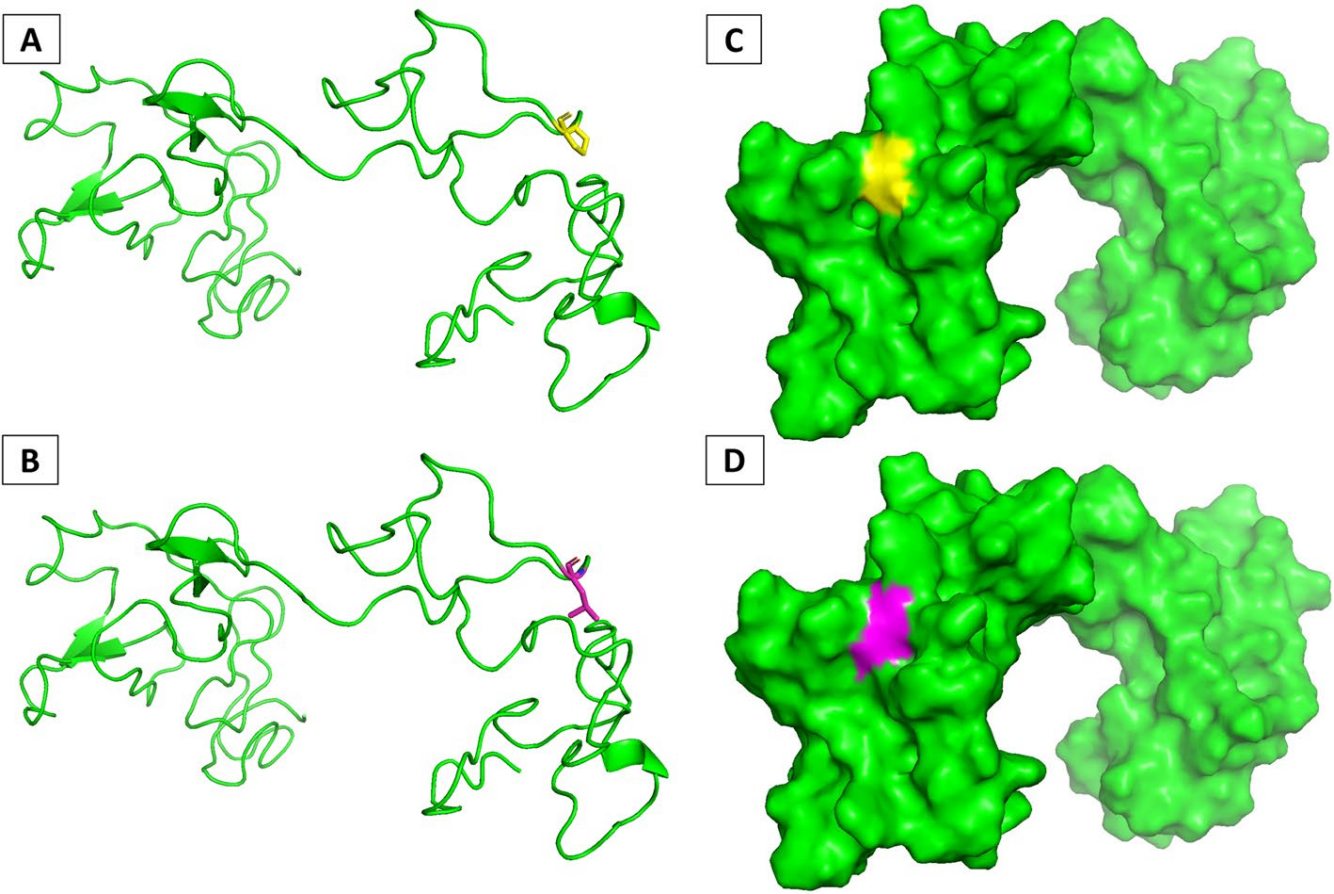
The 3-dimensional structure built through homology model of the domain 3210–3402. **A** wild type protein, in yellow the Proline 3272; **B** mutate protein, in magenta the Leucine; **C** surface wild type protein, in yellow the Proline 3272; **D** surface mutate protein, in magenta the Leucine

To provide new insights into the structure of usherin and the pathological mechanisms caused by small pathogenic in-frame variants, like p.Pro3272Leu, and to add more information about the coding protein to correlate with genotype–phenotype studies, we built in silico 3D structure of the native and mutant protein. Usherin, the product of the *USH2A* gene, is a single-pass transmembrane protein of 5,202 amino acids in humans. The ectodomain of usherin contains a laminin-like-globulin (LGL) domain, 1 N-terminal laminin (LN) domain, 10 epidermal growth factor (LE) domains, 2 globular laminin (LG) domains, and 32 fibronectin III (FN3) domains. The C-terminal intracellular end of usherin has a PDZ binding motif (PBM), which interacts with other USH and deafness proteins, such as whirlin and PDZD7, in retinal photoreceptors and inner ear hair cells. Currently, three-dimensional structure (x-ray, NMR, or single particle cryo-electron microscopy) is not available, because of usherin protein's large size, membrane residence, and potential flexible conformations. To carry out our in-silico studies, we computationally constructed a homology model of the usher protein domain from amino acid 3210 to 3402, where the studied genomic variant is located (P3272L). In addition, the mutated protein was also modelled (Fig. 4).

To understand the impact of this genomic variant on protein structure and function, we applied in-silico methodologies to study and predict the effects of SNPs on protein stability, in particular the free energy change (DDG). All the tools used predicted that the amino acid change from proline to leucine (P3272L) results in a destabilization of the protein with a decrease in DDG. The tools used were DUET, mCSM SDM and CUPSAT with DDGs of -0.212, -0.377, -0.58, -2.51 kcal/mol respectively. Furthermore, the Mupro tool was also used to establish DDG using only the amino acid sequence of all protein. Again, a destabilization was predicted, with DDG = -037 and -0.78, using Vector Machine and Neural Network, respectively.

## Discussion

In this study, we described the clinical and molecular findings of USH patients from three Sardinian families. Molecular analysis showed that the causative pathogenic variant was present in the *USH2A* gene. Although the tissue distribution and subcellular localization of usherin are still debated, there is general agreement that this protein is specifically expressed in retinal photoreceptors and developing cochlear hair cells. Usherin is specifically required in both photoreceptors and cochlear hair cells, where it plays a crucial role cell development and maintenance [26]. Pathogenic variants affecting usherin protein cause failure in stereocilia formation and long-term maintenance of photoreceptors, resulting in sensorineural deafness and visual impairment, the main hallmarks of USH [27].

In mutant mice, with the advance of age photoreceptor degeneration can be detected in the retinas. Mutant mice show progressive shortening of inner/outer photoreceptor segments and thinning of the photoreceptor nuclear layer, leading to the loss of > 50% of photoreceptors and dysregulation of the outer photoreceptor segments, by the age of 20 months. These photoreceptor abnormalities have been associated with the origin of CME in the human retina, detected in 10–50% of RP cases [28]. Similarly, in our study three eyes had CME unresponsive to topical and oral carbonic anhydrase inhibitors.

The p.P3272L variant was first described as a heterozygous compound in various populations (Italian, Dutch, Caucasian, Japanese, Chinese) and only recently in 3 homozygous subjects of Italian origin [23]: one subject had syndromic RP associated with neuro-sensory hearing loss and two patients had non-syndromic RP with different stage of the severity of the disease. According to the published staging criteria [28], the first one was classified as severe (grade 4–5), while the other two as moderate (grade 3).

There are few studies evaluating the impact of pathogenic variants in *USH2A* gene [29, 30], which explored the entire gene structure but not the p.P3272L variant. Sequence alignment of the 32 human usherin FN3 domains showed that 21 of them had the typical β-strands, while F16 and F17 were partial FN3 domains and F3 had a long CD loop. Our pathogenic variant resides in the F16-F17 linker. The model we constructed for the F16-F17 linker [31] shows a U-shaped structure folded in the center at a long loop region. Other pathogenic variants, such as L3145F, C3267R and, C3358Y, reside a few nucleotides away from P3272L and are located at the end of a β-strand or in the middle of loops. Our molecular modeling investigations can contribute to functional studies on pathological mechanisms and therapeutic approaches.

Patients with variants in *USH2A* gene have clinical severity that appears to be related to the gene mutated and the specific type of variant [32]. Homozygosity for some deletions or for duplications in *USH2A* gene has been associated with a severe phenotype [33, 34]. Evidence indicates that, even within the USH2 phenotype, there may be a gradient of severity depending on the specific variant [25, 35].

The patients described in this report have a syndromic phenotype, the severity of which correlates with age. The presence of multiple patients homozygous for the same pathogenic variant with a phenotypic severity correlated with the syndromic form suggests that the Sardinian USH phenotype is the result of a founder pathogenic variant on a specific genomic background. In the specific case of our isolated population, the presence of a founder effect is the most probable explanation for the clinical, phenotypic, and haplotypic homogeneity present in the patients analyzed. The features of genetic and environmental homogeneity that characterize the SardiNIA population certainly help to schematize its perfect segregation with the clinical phenotype. Various founder effects have been described in the Sardinian population for autosomal recessive diseases with haplotypes specific to the Sardinian population associated with individual causative variants, such as in beta Thalassemia [36] or Wilson's disease [37]. As evidence of the prevalence of a founder variant in our population is the significant proportion of asymptomatic heterozygotes carrying this causative variant. For comparison, in the southern European population, the most comparable to ours in terms of genomic characteristics, we found 3 p.P3272L heterozygotes in a total of 5741 individuals (allele frequency = 0.0002613) [14]. We identified in the Sardinian general population, in more than 3500 individuals, a USH heterozygote frequency of 1.89 on which we can hypothesize an ancient founder effect to be related to increased rates of inbreeding and loss of genetic diversity in our patients.

We attempted to estimate the age of the p.P3272L variant using the method proposed by Voight et al., 2006 [38] (data not shown) but results indicated that the variant is too rare to obtain a robust and plausible estimate of haplotype age. We think that, based on heterozygote frequency, it would be possible to suggest a targeted screening of this variant, if not in the general population, at least in families at risk or with familial USH.

## Conclusions

Our results suggest that our approach is effective for the genetic diagnosis of USH. This may lead to (1) a more accurate molecular diagnosis, (2) a better genetic counseling, and (3) an improvement in molecular epidemiology data, critical for future intervention plans.

### Supplementary Information

Supplementary Material 1.

## Data Availability

The datasets used and/or analysed during the current study available from the corresponding author on reasonable request.

## References

[1] Toms M, Pagarkar W, Moosajee M. Usher syndrome: clinical features, molecular genetics and advancing therapeutics. Ther Adv Ophthalmol. 2020;17(12):2515841420952194. 10.1177/2515841420952194. 10.1177/2515841420952194 PMC7502997 32995707

[2] Hagag AM, Mitsios A, Gill JS, et al. Characterisation of microvascular abnormalities using OCT angiography in patients with biallelic variants in USH2A and MYO7A. Br J Ophthalmol. 2020;104(4):480–6. 10.1136/bjophthalmol-2019-314243. 31266775 10.1136/bjophthalmol-2019-314243

[3] Davenport SL, O’Nuallain S, Omenn GS, Wilkus RJ. Usher syndrome in four hard-of-hearing siblings. Pediatrics. 1978;62(4):578–83. 714590 10.1542/peds.62.4.578

[4] Smith RJ, Berlin CI, Hejtmancik JF, et al. Clinical diagnosis of the Usher syndromes. Usher Syndrome Consortium. Am J Med Genet. 1994;50(1):32–8. 10.1002/ajmg.1320500107. 8160750 10.1002/ajmg.1320500107

[5] Smith RJ, Lee EC, Kimberling WJ, et al. Localization of two genes for Usher syndrome type I to chromosome 11. Genomics. 1992;14(4):995–1002. 10.1016/s0888-7543(05)80122-3. 1478678 10.1016/s0888-7543(05)80122-3

[6] Weil D, Blanchard S, Kaplan J, et al. Defective myosin VIIA gene responsible for Usher syndrome type 1B. Nature. 2015;374(6517):60–1. 10.1038/374060a0. 10.1038/374060a0 7870171

[7] Eudy JD, Weston MD, Yao S, et al. Mutation of a gene encoding a protein with extracellular matrix motifs in Usher syndrome type IIa. Science. 1998;280:1753–7. 10.1126/science.280.5370.1753. 9624053 10.1126/science.280.5370.1753

[8] Pakarinen L, Karjalainen S, Simola KO, Laippala P, Kaitalo H. Usher’s syndrome type 3 in Finland. Laryngoscope. 1995;105(6):613–7. 10.1288/00005537-199506000-00010. 7769945 10.1288/00005537-199506000-00010

[9] Zheng QY, Yan D, Ouyang XM, et al. Digenic inheritance of deafness caused by mutations in genes encoding cadherin 23 and protocadherin 15 in mice and humans. Hum Mol Genet. 2005;14(1):103–11. 10.1093/hmg/ddi010. 15537665 10.1093/hmg/ddi010 PMC2858222

[10] Ebermann I, Phillips JB, Liebau MC, et al. PDZD7 is a modifier of retinal disease and a contributor to digenic Usher syndrome. J Clin Invest. 2010;120(6):1812–23. 10.1172/JCI39715. 20440071 10.1172/JCI39715 PMC2877930

[11] Sidore C, Busonero F, Maschio A, et al. Genome sequencing elucidates Sardinian genetic architecture and augments association analyses for lipid and blood inflammatory markers. Nat Genet. 2015;47(11):1272–81. 10.1038/ng.3368. 26366554 10.1038/ng.3368 PMC4627508

[12] Serra R, Rallo V, Pinna A, et al. Polygenic risk score and biochemical/environmental variables predict a low-risk profile of age-related macular degeneration in Sardinia. Graefes Arch Clin Exp Ophthalmol. 2023;261(3):691–8. 10.1007/s00417-022-05858-5. 36264335 10.1007/s00417-022-05858-5 PMC10601990

[13] Sherry ST, Ward MH, Kholodov M, et al. dbSNP: The NCBI database of genetic variation. Nucleic Acids Res. 2001;29(1):308–11. 10.1093/nar/29.1.308. 11125122 10.1093/nar/29.1.308 PMC29783

[14] Karczewski KJ, Francioli LC, Tiao G, et al. The mutational constraint spectrum quantified from variation in 141,456 humans. Nature. 2020;581(7809):434–43. 10.1038/s41586-020-2308-7. 32461654 10.1038/s41586-020-2308-7 PMC7334197

[15] Clarke L, Fairley S, Zheng-Bradley X, et al. The international Genome sample resource (IGSR): a worldwide collection of genome variation incorporating the 1000 Genomes Project data. Nucleic Acids Res. 2017;45(D1):D854–9. 10.1093/nar/gkw829. 27638885 10.1093/nar/gkw829 PMC5210610

[16] Barrett JC, Fry B, Maller J, Daly MJ. Haploview: analysis and visualization of LD and haplotype maps. Bioinformatics. 2005;21(2):263–5. 10.1093/bioinformatics/bth457. 15297300 10.1093/bioinformatics/bth457

[17] Li H. A statistical framework for SNP calling, mutation discovery, association mapping and population genetical parameter estimation from sequencing data. Bioinformatics. 2012;27(21):2987–93. 10.1093/bioinformatics/btr509. 10.1093/bioinformatics/btr509 PMC3198575 21903627

[18] Purcell S, Neale B, Todd-Brown K, et al. PLINK: a tool set for whole-genome association and population-based linkage analyses. Am J Hum Genet. 2007;81(3):559–75. 10.1086/519795. 17701901 10.1086/519795 PMC1950838

[19] Zhang Y. I-TASSER: fully automated protein structure prediction in CASP8. Proteins. 2009;77(Suppl 9):100–13. 10.1002/prot.22588. 19768687 10.1002/prot.22588 PMC2782770

[20] Pires DE, Ascher DB, Blundell TL. DUET: a server for predicting effects of mutations on protein stability using an integrated computational approach. Nucleic Acids Res. 2014;42(Web Server issue):W314–9. 10.1093/nar/gku411. 24829462 10.1093/nar/gku411 PMC4086143

[21] Parthiban V, Gromiha MM, Abhinandan M, Schomburg D. Computational modeling of protein mutant stability: analysis and optimization of statistical potentials and structural features reveal insights into prediction model development. BMC Struct Biol. 2007;7:54. 10.1186/1472-6807-7-54. 17705837 10.1186/1472-6807-7-54 PMC2000882

[22] Cheng J, Randall A, Baldi P. Prediction of protein stability changes for single-site mutations using support vector machines. Proteins. 2006;62(4):1125–32. 10.1002/prot.20810. 16372356 10.1002/prot.20810

[23] Falsini B, Placidi G, De Siena E, et al. USH2A-related retinitis pigmentosa: staging of disease severity and morpho-functional studies. Diagnostics (Basel). 2021;11(2):213. 10.3390/diagnostics11020213. 33535592 10.3390/diagnostics11020213 PMC7912870

[24] Inaba A, Maeda A, Yoshida A, et al. Truncating variants contribute to hearing loss and severe retinopathy in USH2A-associated retinitis pigmentosa in Japanese patients. Int J Mol Sci. 2020;21(21):7817. 10.3390/ijms21217817. 33105608 10.3390/ijms21217817 PMC7659936

[25] Dan H, Huang X, Xing Y, Shen Y. Application of targeted panel sequencing and whole exome sequencing for 76 Chinese families with retinitis pigmentosa. Mol Genet Genomic Med. 2020;8(3):e1131. 10.1002/mgg3.1131. 31960602 10.1002/mgg3.1131 PMC7057118

[26] Liu X, Bulgakov OV, Darrow KN, et al. Usherin is required for maintenance of retinal photoreceptors and normal development of cochlear hair cells. Proc Natl Acad Sci U S A. 2007;104(11):4413–8. 10.1073/pnas.0610950104. 17360538 10.1073/pnas.0610950104 PMC1838616

[27] Iftikhar M, Lemus M, Usmani B, et al. Classification of disease severity in retinitis pigmentosa. Br J Ophthalmol. 2019;103:1595–9. 10.1136/bjophthalmol-2018-313669. 30705041 10.1136/bjophthalmol-2018-313669

[28] Vozzi D, Aaspõllu A, Athanasakis E, et al. Molecular epidemiology of Usher syndrome in Italy. Mol Vis. 2011;17:1662–8. 21738395 PMC3130723

[29] Dong X, Mi LZ, Zhu J, Wang W, Hu P, Luo BH. Alpha(V)beta(3) integrin crystal structures and their functional implications. Biochemistry (Mosc). 2012;51(44):8814–28. 10.1021/bi300734n. 10.1021/bi300734n PMC3495331 23106217

[30] Yu D, Zou J, Chen Q, Zhu T, Sui R, Yang J. Structural modeling, mutation analysis, and in vitro expression of usherin, a major protein in inherited retinal degeneration and hearing loss. Comput Struct Biotechnol J. 2020;18:1363–82. 10.1016/j.csbj.2020.05.025. 32637036 10.1016/j.csbj.2020.05.025 PMC7317166

[31] Eandi CM, Dallorto L, Spinetta R, et al. Targeted next generation sequencing in Italian patients with Usher syndrome: phenotype-genotype correlations. Sci Rep. 2017;7(1):15681. 10.1038/s41598-017-16014-z. 29142287 10.1038/s41598-017-16014-z PMC5688149

[32] Wang F, Wang H, Tuan HF, et al. Next generation sequencing-based molecular diagnosis of retinitis pigmentosa: identification of a novel genotype-phenotype correlation and clinical refinements. Hum Genet. 2014;133(3):331–45. 10.1007/s00439-013-1381-5. 24154662 10.1007/s00439-013-1381-5 PMC3945441

[33] Jiang L, Liang X, Li Y, et al. Comprehensive molecular diagnosis of 67 Chinese Usher syndrome probands: high rate of ethnicity specific mutations in Chinese USH patients. Orphanet J Rare Dis. 2015;10:110. 10.1186/s13023-015-0329-3. 26338283 10.1186/s13023-015-0329-3 PMC4559966

[34] Yang J, Liu X, Zhao Y, et al. Ablation of whirlin long isoform disrupts the USH2 protein complex and causes vision and hearing loss. PLoS Genet. 2010;6(5):e1000955. 10.1371/journal.pgen.1000955. 20502675 10.1371/journal.pgen.1000955 PMC2873905

[35] Pan L, Zhang M. Structures of usher syndrome 1 proteins and their complexes. Physiology (Bethesda). 2012;27(1):25–42. 10.1152/physiol.00037.2011. 22311968 10.1152/physiol.00037.2011

[36] Cao A, Rosatelli C, Pirastu M, Galanello R. Thalassemias in Sardinia: molecular pathology, phenotype-genotype correlation, and prevention. Am J Pediatr Hematol Oncol. 1991;13(2):179–88. 2069229 10.1097/00043426-199122000-00015

[37] Loudianos G, Dessi V, Lovicu M, et al. Molecular characterization of wilson disease in the Sardinian population–evidence of a founder effect. Hum Mutat. 1999;14(4):294–303. 10.1002/(SICI)1098-1004(199910)14:4%3c294::AID-HUMU4%3e3.0.CO;2-9. 10502776 10.1002/(SICI)1098-1004(199910)14:4<294::AID-HUMU4>3.0.CO;2-9

[38] Voight BF, Kudaravalli S, Wen X, Pritchard JK. A map of recent positive selection in the human genome. PLoS Biol. 2006;4(3):e72. 10.1371/journal.pbio.0040072. 16494531 10.1371/journal.pbio.0040072 PMC1382018

