# Supplementary material for: A large-scale screening identified in USH2A gene the P3272L founder pathogenic variant explaining familial Usher syndrome in Sardinia, Italy
Source: BMC Ophthalmol. 2024 Jul 23;24:306. doi: 10.1186/s12886-024-03578-4 (PMC11265335; doi:10.1186/s12886-024-03578-4)
Supplement: Supplementary file 1 — Supplementary Material 1. [file 12886_2024_3578_MOESM1_ESM.pdf]

Supplementary Table S1.
List of all variants identified by whole genome sequencing in the USH2A gene in patients and relatives. The chromosome and position of the variants are depicted according to the human genome assembly, GRCh37/hg19.

Chromosome Position	Reference allele	Alternate allele	rs ID	Gene region	Coding Effect	cDNA change	Protein change	gnomAD Allele Frequency	Polyphen	SIFT
1:215914826	T	C	rs35309576	exonic	missense	NM_206933.4:c.11602A>G	p.Met3868Val	0.2299	benign	tolerated
1:215916563	G	A	rs11120616	exonic	missense	NM_206933.4:c.11504C>T	p.Thr3835Ile	0.2284	benign	tolerated
1:215960167	T	G	rs10864198	exonic	missense	NM_206933.4:c.10232A>C	p.Glu3411Ala	0.5218	benign	tolerated
**1:215972392**	**G**	**A**	**rs764182950**	**exonic**	**missense**	**NM_206933.3:c.9815C>T**	**p.Pro3272Leu**	**0.00004406**	**probably_damaging**	**deleterious**
1:216172380	A	G	rs10864219	exonic	missense	NM_206933.4:c.6506T>C	p.Ile2169Thr	0.5006	benign	tolerated
1:216219781	A	G	rs6657250	exonic	missense	NM_206933.3:c.6317=	p.Ile2106Thr	0.6716	benign	tolerated
1:216258213	A	G	rs56222536	exonic	missense	NM_206933.4:c.4994T>C	p.Ile1665Thr	0.1417	benign	tolerated
1:216348764	C	T	rs1805049	exonic	missense	NM_007123.5:c.4457=	p.Arg1486Lys	0.6241	benign	tolerated
1:216371934	A	C	rs646094	splice region	splicing	NM_206933.4:c.3812-8T>G		0.2335	-	-
1:216465694	G	C	rs35818432	exonic	missense	NM_206933.4:c.1663C>G	p.Leu555Val	0.001738	probably_damaging	deleterious
1:216595306	C	T	rs10779261	exonic	missense	NM_206933.4:c.373G>A	p.Ala125Thr	0.7060	benign	tolerated
